# Comparative responses to nasal allergen challenge in allergic rhinitic subjects with or without asthma

**DOI:** 10.1186/1710-1492-7-8

**Published:** 2011-04-20

**Authors:** Marie-Claire Rousseau, Marie-Eve Boulay, Loie Goronfolah, Judah Denburg, Paul Keith, Louis-Philippe Boulet

**Affiliations:** 1 Centre de recherche, Institut universitaire de cardiologie et de pneumologie de Québec, Québec, QC, Canada; 2 McMaster University, Health Sciences, Hamilton, ON, Canada

## Abstract

**Background:**

Nasal allergen challenge (NAC) is useful to study the pathophysiology of rhinitis, and multiple challenges may more adequately approximate natural exposure.

**Objective:**

To determine the effect of 4 consecutive daily NAC, on clinical and inflammatory parameters in rhinitics with or without asthma.

**Methods:**

Rhinitic subjects were recruited: 19 with mild asthma and 13 without asthma. Subjects underwent a control challenge (normal saline) followed by 4 consecutive daily NAC. Allergen challenge consisted of spraying the chosen allergen extract into each nostril until a positive nasal response occurred. Symptoms were recorded on a Likert scale, and oral peak expiratory and nasal peak inspiratory flows allowed assessment of a nasal blockage index (NBI), for a period of 7 hours. Induced sputum and nasal lavage were performed on control day and after 1 and 4 days of NAC.

**Results:**

Compared with the control day, there was a significant increase in symptom scores and NBI 10 minutes after each last daily NAC in both groups (p < 0.05). Symptom scores and NBI were similar for the 2 groups, except for nasal obstruction and rhinorrhea, which were more marked in subjects with asthma and rhinitis, respectively. Nasal lavage eosinophils were increased after 4 days of challenges in both groups, but there was no change in sputum eosinophils. No cumulative effect or any late response were observed in any of the groups over the challenge period.

**Conclusion:**

Multiple NAC may be a useful tool to study the pathophysiology of allergic rhinitis or its relationships with asthma.

**Trial registration:**

ClinicalTrials.gov NCT01286129

## Background

Asthma and rhinitis are two airway inflammatory diseases that often coexist in the same patient. Up to 80% of asthmatic patients also suffer from allergic rhinitis [1,2] and the risk to develop asthma is almost three times higher among allergic rhinitic subjects compared to controls [3]. Asthma and allergic rhinitis involve common inflammatory mediators that may contribute both to upper and lower airway inflammation [4]. These epidemiological and pathophysiological observations support the concept of the 'United Airways' hypothesis in which upper and lower airways should be considered as a continuum, rather than 2 distinct units [5,6]. However, the mechanisms by which some rhinitic subjects will subsequently develop asthma are still to be understood.

Several techniques have been developed to study the clinical and pathophysiological mechanisms of allergic rhinitis. Among those commonly being used are direct challenges to histamine or allergens, and natural exposure models [7]. Nasal allergen challenge (NAC) is a well-recognized model that has the advantage of reproducing a direct allergen contact in a controlled setting, making possible the use of the same procedure for all subjects with standardized allergens. In comparison with challenges in exposure units, NAC helps to understand specifically the effect of challenging the upper airways on systemic or lower airway inflammation, since the allergen is delivered locally in the nose. This method seems therefore appropriate to study the link between an upper airway disease, such as allergic rhinitis, and a lower airway disease, such as asthma.

Single dose NAC may limit the efficiency of this model, since it may not reproduce the chronicity of a natural allergen exposure. In the past, conflicting results were obtained regarding the impact of upper airway inflammation on the induction of lower airway inflammation using single dose NAC [8,9]. The need to find a model closer to natural allergen exposure has led to the development of repeated allergen challenges [10]. These challenges consist of performing a daily challenge with the chosen allergen and to repeat the procedure over a few consecutive days [10]. This type of challenge has previously been used to investigate the efficiency of different therapies in subjects suffering from seasonal allergic rhinitis [11-13], although it could also be helpful to compare the type of clinical response in allergic rhinitics with or without asthma.

To our knowledge, no studies are available to compare the effect of repeated nasal allergen challenges in non-asthmatic and asthmatic rhinitic subjects. The aim of the present study was therefore to compare the effects of a repeated daily NAC with perennial standardized allergens, on clinical and inflammatory parameters, between allergic rhinitic subjects with or without asthma. This study could also help to compare the nasal response of these 2 groups in regard to a possible cumulative effect, the presence of a late response and the type of response.

## Methods

### Subjects

Thirty-two non-smoking subjects were recruited: 19 had mild stable asthma associated with allergic rhinitis (A) and 13 had allergic rhinitis without asthma (R). Rhinitis was defined according to the ARIA guidelines [14]. All subjects had a positive reaction to cat hair and/or house dust mite (*Dermatophagoides pteronyssinus*) aeroallergens on allergy skin prick tests and reported rhinitis symptoms when exposed to an environment containing this allergen. Asthma was defined according to the criteria proposed by the American Thoracic Society (ATS) [15]. At entry into the study, all subjects had baseline forced expiratory volume in one second (FEV_1_) >70% predicted. Asthmatic subjects had a provocative concentration of methacholine causing a 20% fall in FEV_1 _(PC_20_) ≤ 16 mg/mL and non-asthmatic subjects had a PC_20 _>16 mg/mL.

Subjects who had received oral or inhaled corticosteroids in the past 6 months, nasal corticosteroids in the past 3 months, and anti-inflammatory or antihistamine drugs in the past 7 days were excluded from the study. Asthmatic subjects did not use any rescue medication 7 hours prior to each visit and 7 hours following every challenge. None of the subjects experienced upper or lower respiratory tract infection within one month preceding the beginning of the study. All subjects provided a written informed consent and the study was approved by the institutional Ethics Committees (Institut universitaire de cardiologie et de pneumologie de Québec and McMaster University).

### Study design

**Figure 1:**
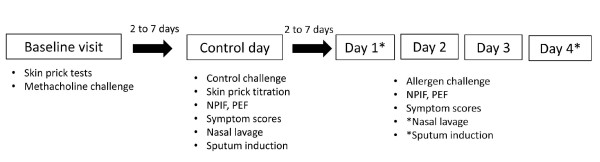
**Study design**. The protocol was divided into 3 different parts: a baseline visit, a control day (nasal challenge with 0.9% saline) and 4 consecutive days of nasal allergen challenge (days 1-4).

The study design is presented in Figure 1. The study was performed outside the pollen season. On a baseline visit, 2 to 7 days prior to control challenge, allergy skin prick tests and methacholine inhalation challenge were done. Subsequent to baseline visit, a control challenge was done, followed, a week later, by repeated NACs. NACs were performed over 4 consecutive days, in the morning. Nasal peak inspiratory flows (NPIF), oral peak expiratory flows (PEF), and symptoms were recorded at baseline and at regular intervals over 7 hours post-challenge on each challenge day. Induced sputum and nasal lavage specimen were obtained 7 hours following the control challenge and the first and last NAC.

### Skin prick tests and titration

Atopy was determined using skin prick tests procedure for common aeroallergens. Normal saline and histamine were used as negative and positive controls, respectively. Skin wheal diameter was recorded at 10 minutes as the mean of 2 perpendicular measurements. A positive response was defined as a skin wheal diameter of 3-mm or more compared to negative control. The choice of the allergen for NAC, either cat hair or *D. pteronyssinus*, was based upon the intensity of the sensitization, determined by skin prick tests, and questions to the subjects about their rhinitis symptoms to these allergens.

Skin prick titration was done prior to allergen challenge in order to determine the starting allergen concentration for NAC. The titration was completed in the same way as for skin prick tests, but using a series of dilutions of the chosen allergen. The procedure was done in duplicate. The concentration that yielded a skin prick test of 2-mm minimum was the chosen starting concentration for nasal challenge.

### Spirometry and methacholine inhalation challenge

Baseline FEV_1 _and forced vital capacity (FVC) were measured according to the ATS criteria [16] and predicted values were obtained from Knudson [17]. Methacholine bronchial challenge was done as described by Juniper [18].

### Nasal challenge

NAC was performed as previously described by Wilson *et al. *[9] using perennial allergens (cat hair, 10,000 BAU/mL or *D. pteronyssinus*, 30,000 AU/mL; Omega Laboratories, Montreal, QC, Canada). Briefly, the nasal control challenge was done using 4 exposures of 0.9% saline at 10 min intervals in the same way as for allergens. Nasal allergen challenge was done using tenfold increasing concentrations of the allergen extract chosen, either cat hair or *D. pteronyssinus*, beginning with the concentration pre-determined by skin titration. Before spraying, subjects were asked to inhale through their mouth to total lung capacity and to hold their breath, in order to avoid lower airway contamination by the test agent [8,19]. Then, one squirt (0.1mL) of the starting concentration was sprayed into each nostril from a metered-dose pump spray (Aventis Pharma, Laval, QC, Canada).

Symptom scores derived from: blockage 0-2 (absence = 0, moderate = 1, severe = 2), secretion 0-2 (absence = 0, moderate = 1, severe = 2), sneezing 0-2 (< 3 sneezes = 0, 3-5 sneezes = 1, > 5 sneezes = 2), itching eye or throat 0-1 (absence = 0, presence = 1), and conjunctivitis, cough, urticaria or dyspnea 0-1 (absence = 0, presence = 1), were recorded 10 minutes after each provocation. The total score of symptoms was calculated by adding the scores up to a maximum score of 8. The procedure was repeated with tenfold increasing concentrations until the highest concentration was given or a positive response occurred. A positive response was achieved when the total score of symptoms reached a minimum of 3 points. If this was not obtained with the highest concentration, then the dose was increased by giving 2 squirts and, if necessary, 3 squirts in each nostril.

Nasal obstruction was measured quantitatively using NPIF and PEF before provocation and at determined time-points for 7 hours post-provocation. At these same time-points, subjects evaluated the intensity of their symptoms for nasal obstruction, rhinorrhea, sneezing, nasal itching, and cough. A score was given for each of these symptoms, using a 7-point Likert scale, graduated from 0 = Not troubled, to 6 = Severely troubled.

### Peak Flows

NPIF was measured with a nasal peak flow meter (In Check, Clement-Clarke International Ltd, Harlow, Essex, UK), using the method previously described by Youlten [20]. The best of three measurements was recorded. The use of NPIF and PEF (Mini Wright Peak Flow Meter, Clement-Clarke) allowed obtaining the nasal blockage index (NBI), using a modified equation from Taylor *et al. *[21]:

### Nasal Lavage

Nasal lavage was performed as described by Cormier *et al. *[22]. Briefly, subjects were in a sitting position with the neck flexed at 45° from horizontal. Subjects were asked to blow their nose before 5 mL of phosphate buffered saline (PBS) solution were instilled into each nostril with a needleless syringe. Subjects then flexed the neck and expelled nasal lavage fluid into a sterile dish. Throughout the procedure, subjects were asked to refrain from breathing or swallowing. Lavage fluid was filtered and centrifuged. Supernatant was aliquoted and frozen until further analyses. Cells were resuspended and counted to determine total cell count and viability. Slides were then prepared and stained with Diff-Quik for differential cell count.

### Induced Sputum

Sputum induction was performed using the method described by Pin *et al. *[23] and modified by Pizzichini *et al. *[24]. Sputum was processed within 2 hours following induction. Briefly, mucus plugs were selected from saliva, weighed, treated with 4 times their volume of dithiothreitol (DTT) and rocked for 15 minutes. The reaction was stopped with an equal volume of Dulbecco's phosphate buffered saline (D-PBS) 1X, filtered and counted to determine total cell count and viability. Suspension was adjusted to 1 × 10^6 ^cells/mL and 2 slides were prepared and stained with Diff-Quik for differential cell count. Following centrifugation, sputum supernatants were aliquoted and frozen.

### Mediator measurements

The presence of eosinophilic cationic protein (ECP) in nasal lavage and induced sputum supernatants was measured by ELISA (Measacup ECP, MBL International Corporation, Woburn, MA) according to manufacturer's instructions. Nasal lavage samples were processed non-diluted and sputum samples were diluted 1:75. The detection limit of the assay was 0.125 ng/mL.

### Statistical Analysis

Values are reported as mean ± SEM. Two different statistical procedures were completed 1) to compare asthmatic to rhinitic subjects over a time course at specific visits, 2) to compare asthmatic to rhinitic subjects over a time course from different visits. 1) We considered subjects as random block effects. For each visit, values were measured at time 0, 10, 20, 30, 45 min, 1h, 1.5h, 2h, 3h, 4h, 5h, 6h, and 7h. The statistical approach used was to perform a three-way repeated measures design where group and time were analysed as fixed factors. A symmetric component variance-covariance structure was defined to analyse repeated measurements as time points were not equally spaced. The multivariate normality was verified using Mardia's test. 2) We considered subjects as random block effects. The statistical approach used was to perform a four-way doubly-repeated measures design where group, visit, and time were analysed as fixed factors. The unstructured compound symmetry structure was used to analyse repeated measurements. Tukey's comparisons were performed to compare visits and time points. The multivariate normality was verified using Mardia's test. The results were considered significant with p-values ≤ 0.05. The data were analysed using the statistical package program SAS v9.1.3 (SAS Institute Inc., Cary, NC)

## Results

### Subjects

The characteristics of the subjects are presented in Table 1. Age and baseline FEV_1 _were similar between the 2 groups. Lower initial dilutions of allergen given for challenge were used for asthmatics compared to rhinitics. Allergens used for challenge were equally distributed within and between groups.

**Table 1:** Characteristics of subjects

n	Rhinitics 13	Asthmatics 19
*Age (years)	24 (19-32)	24 (19-41)
**Gender (M: F)	7: 6	5: 14
**Allergen used for NAC (Cat hair: *D.pteronyssinus*)	5 : 8	11 : 8
**Initial dilution given (non-diluted, 1:10, 1:100, 1:1000)	1, 5, 2, 5	0, 3, 5, 11
*FEV_1 _(% predicted)	108 (87-125)	103 (87-125)

*Data are presented as mean (range)

** Data are presented as number of subjects

FEV_1_: Forced expiratory volume in one second

### Nasal blockage index

**Figure 2:**
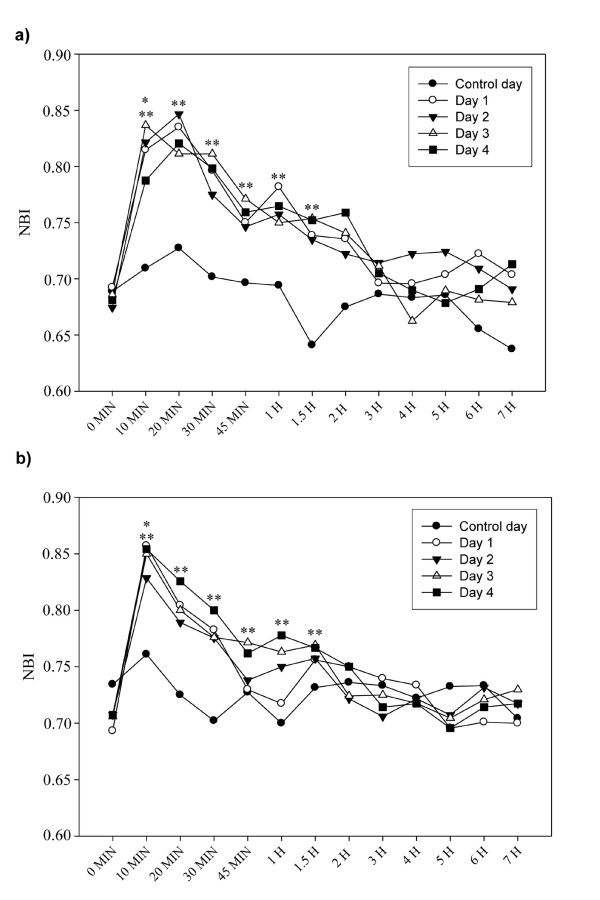
**Effect of nasal challenge with saline (control day) or allergen (days 1-4) on NBI**. **(a) **for rhinitics and **(b) **for asthmatics at 0 min and over 7 hours post-challenge. *p < 0.05; 0 min vs 10 min on days 1-4. **p < 0.05; Control day vs days 1-4.

Over the 4 challenge days, no differences in baseline NBI values were detected between and within subjects, irrespective of their group (Figure 2). On control day, no significant change in NBI was observed over time and the response was similar between groups. Ten minutes after obtaining a positive response on each allergen challenge day, an increased NBI value was observed for the two groups compared with baseline value (p < 0.05) and the response was similar for the 2 groups. Moreover, the comparison of each allergen challenge day with control day showed a significant increase in NBI from 10 min to 1.5h post-challenge.

### Symptom scores

All subjects recorded their symptoms for a 7-hour period post-challenge. In regard to nasal obstruction score, on control day, no symptoms were observed for any of the 2 groups. When comparing each allergen challenge day with control day, the score remained significantly increased until 1.5h post-challenge for the 2 groups (p < 0.05). Overall, asthmatic subjects had a higher nasal obstruction score than rhinitics (p = 0.04).

No symptom of rhinorrhea was observed on control day in any of the 2 groups, while a significant increase was observed until one hour post-challenge on each allergen challenge day, in comparison with control day, for both rhinitics and asthmatics (p < 0.05). Overall, subjects with rhinitis alone had a higher rhinorrhea score than those with rhinitis and asthma (p = 0.03).

The nasal itching score was significantly increased until one hour following allergen challenge for rhinitics and for 30 minutes following challenge for asthmatics, compared with control day. Overall, no significant difference was observed between groups (p > 0.05).

No significant change for sneezing and cough symptom scores were observed on any of the 4 allergen challenge days in the two groups, compared with control day. However, we observed that a limited number of subjects experienced cough symptoms at least at one time-point on allergen challenge days (A = 9/19 (47%) and R = 7/13 (54%)).

No late response was observed in any of the two groups during the challenge period.

### Upper and lower airway inflammation

Data for changes in inflammatory parameters after 1 and 4 days of nasal allergen challenges are presented in Table 2. There was a significant increase in the percentage of eosinophils in nasal lavage after 4 days of nasal allergen challenges in rhinitics and asthmatics compared with control challenge (p < 0.05). The levels of ECP in nasal lavage were significantly increased after 1 day of nasal allergen challenge in both groups (p < 0.05), but not after 4 days. There was no inflammatory change in the percentage of eosinophils and in ECP levels in induced sputum after both the first and last allergen challenges compared with control challenge.

**Table 2:** Inflammatory parameters following nasal control challenge, and 1 and 4 days of nasal allergen challenges

Parameter	Rhinitics			Asthmatics		
	Control	Day 1	Day 4	Control	Day 1	Day 4
Nasal lavage eosinophils (%)	1.3 ± 0.9	3.0 ± 1.3	15.5 ± 9.6 *	2.1 ± 0.6	7.5 ± 4.3	15.7 ± 5.4*
Nasal lavage ECP (ng/mL)	3.8 ± 1.5	8.7 ± 3.4 *	7.9 ± 2.6	8.6 ± 3.2	10.3 ± 2.7 *	9.4 ± 4.0
Induced sputum eosinophils (%)	2.0 ± 1.3	1.3 ± 1.5	1.6 ± 1.0	5.6 ± 1.8	6.0 ± 1.7	4.1 ± 1.4
Induced sputum ECP (ng/mL)	72 ± 20	112 ± 41	98 ± 36	146 ± 43	171 ± 41	242 ± 106

Data are presented as mean ± SEM

* p < 0.05 vs control challenge

## Discussion

Nasal challenges performed on a single occasion may not represent accurately a natural allergen exposure, leading to the development of multiple challenges, done over a few consecutive days, which could be more representative of the reality. This type of nasal challenge has been used in few studies in the past [11-13,25]. In these, only allergic rhinitic subjects were recruited and three out of four were done to compare the effect of different types of rhinitis medications [11-13]. To our knowledge, the present study is the first to compare the effects of multiple NAC in rhinitic subjects with or without asthma.

One objective of this study was to determine how allergic rhinitic subjects with or without asthma would react following a multiple NAC, regarding the type and duration of induced symptoms. Our results showed that the two groups responded in the same way, except for nasal obstruction and rhinorrhea symptoms. Asthmatics were more likely to report nasal obstruction, whereas rhinitics had more symptoms of rhinorrhea. This is of interest, since nasal obstruction may lead to mouth breathing, allowing an increased quantity of allergens to penetrate into the lower airways, inducing inflammation, and potentially triggering asthma symptoms.

The other objective was to compare the inflammatory response of allergic rhinitic subjects with or without asthma following a repeated nasal allergen challenge. We observed a significant increase in nasal lavage ECP concentrations in both groups after 1 day of challenge, which was no more significant after 4 days of challenge. Furthermore, an increase in upper airway eosinophils after 4 days of challenge was observed in both groups. No significant difference in upper airway inflammation was observed between groups. We did not observe a significant change in lower airway inflammation following neither the first nor the last allergen challenge, determined by sputum eosinophils and ECP. There was no significant difference in lower airway inflammation between groups. However, since upper airway inflammation appeared only at the last challenge day, we think that it could be of interest to continue this type of challenge over a few more days to be able to induce lower airway inflammation by stimulating upper airways, and possibly observe a different inflammatory profile between groups.

We used perennial allergens (cat hair and *D. pteronyssinus*) to perform allergen challenges since these allergens are more associated with lower airway hyperreactivity [13] and lower airway inflammation than outdoor ones [26]. Therefore, it is of interest to observe the effect of upper airway challenge with perennial allergens on lower airway symptoms given that, to our knowledge, no study used perennial allergens to perform multiple nasal allergen challenges. A limited number of subjects experienced cough symptoms following nasal challenge, reflecting the link between upper airway stimulation and lower airway symptoms. Further studies are needed to determine if rhinitic subjects experiencing these symptoms are more at risk to develop asthma.

Several techniques have been used to deliver allergens to the nose [27]. In our study, the nasal pump spray technique was used for two main reasons. First, it has the advantage of delivering the allergen over the entire nasal mucosa, instead of a localized area, as it can be observed, for example, with pipettes or paper discs [28]. Second, we know the exact quantity of solution sprayed into the nose. With the pump spray delivery method, no allergen should penetrate in the lower airways if the subjects previously inhaled to total lung capacity and held their breath before spraying the solution [19]. We believe that the results obtained in our study are the specific consequences of upper airway stimulation.

The challenge was repeated over 4 consecutive mornings allowing to determine if there was a priming effect. This effect was first described by Connell as the ability to use smaller amounts of allergen in subsequent challenges to induce the same or greater degree of symptomatic allergic response [29,30]. This observation was then confirmed by others [31]. However, although this concept is now well accepted, it seems that repeated allergen challenge and priming are not necessarily linked [10]. Several factors play a role in nasal priming, one of which is the way the response is recorded. The strongest evidence of priming comes from changes in mediator levels and inflammatory cell numbers in the nose, which do not always coincide with physiological or clinical changes. In our study, when looking at symptoms scores or NBI results, this effect was not observed between the 4 days of challenge, in any of the two groups. However, in both groups, we did observe an increase in nasal lavage eosinophils over the study period that reached significance at day 4 of challenge. ECP levels also significantly increased on the first day of challenge compared with control day in both groups, but no further increase was observed at day 4, although levels were still higher. This is suggestive of priming at the immunological level, as also shown by McDermott *et al.*, who performed repeated allergen challenge over 8 consecutive days, recording symptoms scores and collecting samples at day 2 and 24h following the last challenge (day 9) [32]. In that study, they did not observe further increase in symptoms scores between day 2 and day 9 of allergen challenge, but reported an additional increase in IL-5 and a decrease in IFN-γ at day 9 compared with day 2. In addition, as suggested by Wachs *et al.*, a priming response may be observed overall, but there is a large variety in individual response patterns to repeated allergen provocation [25].

We did not observe the development of a late nasal or bronchial response in the hours following the challenges, even on the last provocation day. There is a lot of variability in late nasal allergic response prevalence ranging between 30% and 50% [33]. The intensity of the immediate reaction cannot be considered to be a suitable predictor of the late response [33]. Various factors such as the differences in challenge procedure, the data recording techniques and the cut-offs for positivity can be involved, although the mechanisms have not been fully clarified [33]. In the present study, subjects were recording their symptoms scores and NPIF hourly, until 7 hours post challenge. Late responses can be observed between 3 and 8 hours post exposure to the allergen. An extension in the collection of data over 7 hours post-challenge could have allowed to observe a late response in some subjects, although unlikely. We observed an increase in nasal lavage eosinophils only following 4 days of challenge, but it is possible that the inflammatory response was not strong enough to induce a late increase in nasal symptoms or a significant decrease in NPIF.

To be sure that the results were not influenced by outdoor allergens, subjects sensitized to seasonal allergens were tested out of the pollen season. We are aware that some indoor allergens, such as dust mites, cannot be avoided completely. In this regard, we asked the subjects to keep their life habits as stable as possible throughout the study. In addition, we compared the allergen challenge results with the control challenge results, which were done in the same way. These precautions helped to better assess the specific effect of the allergens tested, independently of the presence of perennial allergens in the subjects' environment. However, we cannot exclude the possibility of interference of such continuous exposure to perennial allergens with the clinical response to allergen challenge. Indeed, Reinartz *et al. *showed that subjects mono-sensitized to grass pollen had lower nasal symptom scores and NPIF following nasal challenge than subjects mono-sensitized to HDM or poly-sensitized subjects [34]. This could not be explained by serum levels of total or specific IgE, suggesting that altered local immune-regulatory processes could be involved. However, the influence of pattern or sensitization on the late-phase response was not studied. In addition, the same dose of allergen was administered to all subjects while some subjects might have needed a lower dose to induce an early response. Since cat hair and HDM are known to be potent inducers of the late response in bronchial allergen provocations and as we induced a significant upper airways clinical response, it is unlikely that the choice of allergen is responsible for the lack of late response in this study.

## Conclusions

This study shows that multiple nasal challenges with perennial allergens induce more rhinorrhea in rhinitic subjects without asthma and more nasal obstruction in rhinitic subjects with asthma, suggesting a different symptomatic profile between these 2 groups. We found no evidence of cumulative effect or late response after multiple nasal challenges in both groups.

In conclusion, we think that this method could be useful to assess the effect of treatment on symptoms. However, future studies are needed to improve this protocol of repeated nasal allergen challenge to induce lower airway inflammation, maybe by extending the challenge period or increasing the doses given.

## Abbreviations

A: Allergic rhinitis with asthma; ATS: American thoracic society; DTT: Dithiothreitol; ECP: Eosinophil cationic protein; FEV_1_: Forced expiratory volume in one second; FVC: Forced vital capacity; HDM: House-dust mite; IFN-γ: Interferon gamma; IL-5: Interleukin-5; NAC: Nasal allergen challenge; NBI: Nasal blockage index; NPIF: Nasal peak inspiratory flow; PC_20_: Provocative concentration of methacholine inducing a 20% decrease in FEV1; PEF: peak expiratory flow; R: Allergic rhinitis without asthma.

## Competing interests

MCR, MEB, LG have no competing interests.

PK competing interests are:

Advisory Boards and Lecture Fees: GlaxoSmith Kline, Merck, Nycomed.

Research funding for participating in multicenter studies: Affexa Life Sciences, Allergy Therapeutics, GlaxoSmithKline, Merck, Nycomed.

JD was the recipient of grants from AllerGen NCE Inc. and CIHR and is the CEO and Scientific Director of AllerGen NCE.

LPB competing interests are:

**Advisory Boards**: AstraZeneca, Altana, GlaxoSmithKline, Merck Frosst and Novartis.

**Lecture fees**: 3M, Altana, AstraZeneca, GlaxoSmithKline, Merck Frosst and Novartis.

**Sponsorship for investigator-generated research**: AstraZeneca, GSK, Merck Frosst, Schering

**Research funding for participating in multicenter studies**: 3M, Altana, AsthmaTx, AstraZeneca, Boehringer-Ingelheim, Dynavax, Genentech, GlaxoSmithKline, IVAX, MedImmune, Merck Frosst, Novartis, Roche, Schering, Topigen, Wyeth.

**Support for the production of educational materials**: AstraZeneca, GlaxoSmithKline and

Merck Frosst.

**Governmental**: Adviser for the Conseil du Médicament du Québec Member of the Quebec Workmen Compensation Board Respiratory Committee

**Organisational**: Chair of the Canadian Thoracic Society Guidelines Dissemination and Implementation Committee. Co-leader of the Therapeutics Theme of the Canadian AllerGen Network of Centers of Excellence. Holder of the Laval University Chair on knowledge Transfer, Prevention and Education in Respiratory and Cardiovascular Health. Member of the asthma committee of the World Allergy Organisation.

## Authors' contributions

MCR participated in the conception and design of the study, in the generation, analysis and interpretation of the data, and drafted the manuscript. MEB conceived, designed and coordinated the study and was involved in the generation, analysis and interpretation of the data as well as in the preparation and critical revision of the manuscript. LG participated to the data generation, analysis and interpretation of the data as well as preparation and critical revision of the manuscript. JD participated in the conception and design of the study and in preparation and critical revision of the manuscript. PK participated in the conception and design of the study, analysis and interpretation of the data, and preparation and critical revision of the manuscript. LPB was involved in the conception and design of the study, analysis and interpretation of the data, and preparation and critical revision of the manuscript. All authors approve the final version of the manuscript.

## References

[B1] Bugiani M Carosso A Migliore E Piccioni P Corsico A Olivieri M Ferrari M Pirina P de Marco R Allergic rhinitis and asthma comorbidity in a survey of young adults in Italy Allergy 2005 60 165 170 10.1111/j.1398-9995.2005.00659.x 15647036

[B2] Leynaert B Neukirch C Liard R Bousquet J Neukirch F Quality of life in allergic rhinitis and asthma Am J Respir Crit Care Med 2000 162 1391 1396 11029350 10.1164/ajrccm.162.4.9912033

[B3] Settipane RJ Hagy GW Settipane GA Long-term risk factors for developing asthma and allergic rhinitis: a 23-year follow-up study of college students Allergy Proc 1994 15 21 25 10.2500/108854194778816634 8005452

[B4] Grossman J One airway, one disease Chest 1997 111 11S 16S 10.1378/chest.111.2_Supplement.11S 9042022

[B5] Togias A Rhinitis and asthma: evidence for respiratory system integration J Allergy Clin Immunol 2003 111 1171 1183 10.1067/mai.2003.1592 12789212

[B6] Rowe-Jones JM The link between the nose and lung, perennial rhinitis and asthma- is it the same disease? Allergy 1997 52 suppl.36 20 28 9212859 10.1111/j.1398-9995.1997.tb04818.x

[B7] Day JH Ellis AK Rafeiro E Ratz JD Briscoe MP Experimental models for the evaluation of treatment of allergic rhinitis Ann Allergy Asthma Immunol 2006 96 263 277 10.1016/S1081-1206(10)61235-5 16498847

[B8] Braunstahl GJ Overbeek SE Kleinjan A Prins JB Hoogsteden HC Fokkens WJ Nasal allergen provocation induces adhesion molecule expression and tissue eosinophilia in upper and lower airways J Allergy Clin Immunol 2001 107 469 476 10.1067/mai.2001.113046 11240947

[B9] Wilson AM Duong M Crawford L Denburg J An evaluation of peripheral blood eosinophil/basophil progenitors following nasal allergen challenge in patients with allergic rhinitis Clin Exp Allergy 2005 35 39 44 10.1111/j.1365-2222.2004.02072.x 15649264

[B10] de Bruin-Weller MS Weller FR De Monchy JG Repeated allergen challenge as a new research model for studying allergic reactions Clin Exp Allergy 1999 29 159 165 10.1046/j.1365-2222.1999.00434.x 10051718

[B11] Ahlstrom-Emanuelsson C Persson CG Svensson C Andersson M Hosszu Z Akerlund A Greiff L Establishing a model of seasonal allergic rhinitis and demonstrating dose-response to a topical glucocorticosteroid Ann Allergy Asthma Immunol 2002 89 159 165 10.1016/S1081-1206(10)61932-1 12197572

[B12] Andersson M Svensson C Persson C Akerlund A Greiff L Dose-dependent effects of budesonide aqueous nasal spray on symptoms in a daily nasal allergen challenge model Ann Allergy Asthma Immunol 2000 85 279 283 10.1016/S1081-1206(10)62530-6 11061470

[B13] Korsgren M Andersson M Borga O Larsson L den-Raboisson M Malmqvist U Greiff L Clinical efficacy and pharmacokinetic profiles of intranasal and oral cetirizine in a repeated allergen challenge model of allergic rhinitis Ann Allergy Asthma Immunol 2007 98 316 321 10.1016/S1081-1206(10)60876-9 17458426

[B14] Bousquet J van Cauwenberge P Khaltaev N Allergic rhinitis and its impact on asthma J Allergy Clin Immunol 2001 108 S147 S334 10.1067/mai.2001.118891 11707753

[B15] ATS statement. Standardization of spirometry-1987 update Am Rev Respir Dis 1987 136 1285 1298 3674589 10.1164/ajrccm/136.5.1285

[B16] Miller MR Hankinson J Brusasco V Burgos F Casaburi R Coates A Crapo R Enright P van der Grinten CP Gustafsson P Jensen R Johnson DC MacIntyre N McKay R Navajas D Pedersen OF Pellegrino R Viegi G Wanger J Standardisation of spirometry Eur Respir J 2005 26 319 338 10.1183/09031936.05.00034805 16055882

[B17] Knudson RJ Lebowitz MD Holberg CJ Burrows B Changes in the normal maximal expiratory flow-volume curve with growth and aging Am Rev Respir Dis 1983 127 725 734 6859656 10.1164/arrd.1983.127.6.725

[B18] Juniper EF Cockcroft DW Kolendowicz R Histamine and methacholine inhalation test: a laboratory tidal breathing protocol Astra Draco AB 1994 1 49

[B19] Corren J Adinoff AD Irvin CG Changes in bronchial responsiveness following nasal provocation with allergen J Allergy Clin Immunol 1992 89 611 618 10.1016/0091-6749(92)90329-Z 1740589

[B20] Youlten LJF The peak nasal inspiratory flow meter: a new instrument for the assessment of the response to immunotherapy in seasonnal allergic rhinitis Allergol Immunopathol 1980 8 344

[B21] Taylor G Macneil AR Freed DL Assessing degree of nasal patency by measuring peak expiratory flow rate through the nose J Allergy Clin Immunol 1973 52 193 198 10.1016/0091-6749(73)90057-2 4270244

[B22] Cormier Y Laviolette M Bedard G Dosman J Israel-Assayag E Effect of route of breathing on response to exposure in a swine confinement building Am J Respir Crit Care Med 1998 157 1512 1521 9603131 10.1164/ajrccm.157.5.9707113

[B23] Pin I Gibson PG Kolendowicz R Use of induced sputum cell counts to investigate airway inflammation in asthma Thorax 1992 47 25 29 10.1136/thx.47.1.25 1539140 PMC463545

[B24] Pizzichini E Pizzichini MMM Efthimiadis A Evans S Morris MM Squillace D Gleich GJ Dolovich J Hargreave FE Indices of airway inflammation in induced sputum: reproducibility and validity of cell and fluid-phase measurements Am J Respir Crit Care Med 1996 154 308 317 8756799 10.1164/ajrccm.154.2.8756799

[B25] Wachs M Proud D Lichtenstein LM Kagey-Sobotka A Norman PS Naclerio RM Observations on the pathogenesis of nasal priming J Allergy Clin Immunol 1989 84 492 501 10.1016/0091-6749(89)90362-X 2477429

[B26] Boulay ME Boulet LP Influence of natural exposure to pollens and domestic animals on airway responsiveness and inflammation in sensitized non-asthmatic subjects Int Arch Allergy Immunol 2002 128 336 343 10.1159/000063856 12218372

[B27] Andersson M Greiff L Svensson C Persson C Various methods for testing nasal responses in vivo: a critical review Acta Otolaryngol 1995 115 705 713 10.3109/00016489509139391 8749189

[B28] Litvyakova LI Baraniuk JN Nasal provocation testing: a review Ann Allergy Asthma Immunol 2001 86 355 364 10.1016/S1081-1206(10)62478-7 11345277

[B29] Connell JT Quantitative intranasal pollen challenge. II. Effect of daily pollen challenge, environmental pollen exposure, and placebo challenge on the nasal membrane J Allergy 1968 41 123 139 10.1016/0021-8707(68)90053-1 5238591

[B30] Connell JT Quantitative intranasal pollen challenges. 3. The priming effect in allergic rhinitis J Allergy 1969 43 33 44 10.1016/0021-8707(69)90018-5 5249197

[B31] Sahin-Yilmaz AA Naclerio RM John T. Connell and nasal priming J Allergy Clin Immunol 2006 118 1190 1192 10.1016/j.jaci.2006.04.006

[B32] McDermott RA Nelson HS Dreskin SC Mediator measurements after daily instillation of allergen: Increased IL-5 and decreased IFN-gamma Allergy Asthma Proc 2008 29 146 151 10.2500/aap.2008.29.3094 18430311

[B33] Pelikan Z Late nasal response (LNR) - its clinical characteristics, feature, and possible mechanism(s) 1990 111 155

[B34] Reinartz SM van Ree R Versteeg SA Zuidmeer L van Drunen CM Fokkens WJ Diminished response to grass pollen allergen challenge in subjects with concurrent house dust mite allergy Rhinology 2009 47 192 198 19593978

